# An antibiotic recipe for an arrhythmic disaster

**DOI:** 10.5830/CVJA-2015-006

**Published:** 2015

**Authors:** Keir McCutcheon, Pravin Manga

**Affiliations:** Division of Cardiology, Department of Medicine, University of the Witwatersrand, and Charlotte Maxeke Academic Hospital, Johannesburg, South Africa; Division of Cardiology, Department of Medicine, University of the Witwatersrand, and Charlotte Maxeke Academic Hospital, Johannesburg, South Africa

**Keywords:** erythromycin, QT prolongation, complete heart block, pacing, torsade de pointes

## Abstract

We describe the case of a patient who developed torsade de pointes during temporary pacemaker insertion after administration of intravenous erythromycin. The case highlights the dangers of administering drugs that prolong the QT interval in patients with complete atrioventricular block, and we discuss the underlying pathophysiological recipe that can lead to a potential arrhythmic disaster.

## Abstract

Torsade de pointes (TdP), a polymorphic ventricular tachycardia caused by dispersion of depolarisation within the ventricles, is an important complication of atrioventricular (AV) conduction disorders.^1^ Various classes of drugs including antimicrobials, antiarrhythmic and psychotropic drugs may lead to prolongation of the QT interval with an increased risk of TdP, especially in patients with other risk factors for QT prolongation. However, to date, there have been very few reports of TdP due to drugs in patients with AV block,^2^,^3^ and, to our knowledge, no case reports highlighting the hazard of using erythromycin in patients with complete AV block.

The QT interval, which shortens during tachycardia and lengthens during bradycardia, is the most useful measure to predict a patient’s risk of developing TdP and several formulae are available to correct for heart rate, the commonest being Bazzet’s formula.^4^ Women normally have slightly longer QT intervals than men.

We describe here a case of TdP during pacemaker implantation after erythromycin administration. The case highlights the potentially life-threatening effects of prescribing QT-prolonging drugs in patients with severe bradyarrhythmias.

## Case report

A 68-year-old woman, with a background of hypertension controlled on medical therapy, presented with vague symptoms of fatigue and poor exercise tolerance. She had no history of antibiotic, anti-arrhythmic or psychotropic drug use in the previous month. She had no history or family history of syncope or sudden cardiac death and had no other significant past medical history. However, she reported having an allergy to penicillin.

**Figure 1.**
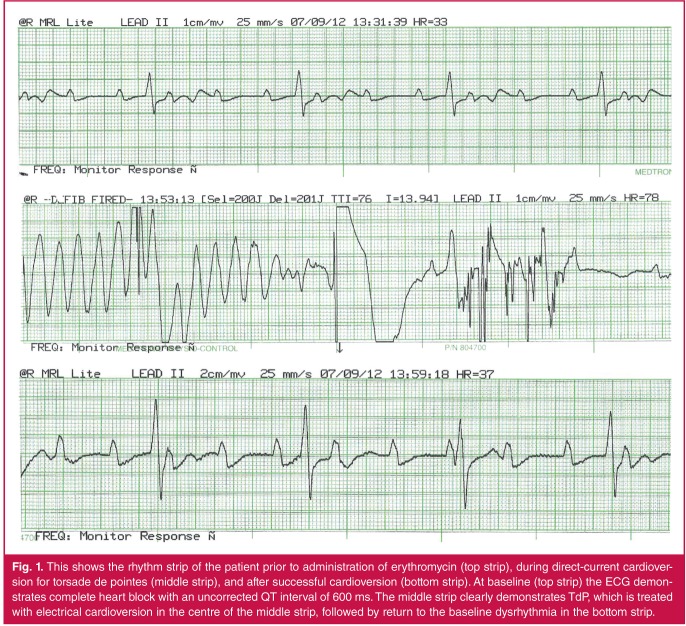
This shows the rhythm strip of the patient prior to administration of erythromycin (top strip), during direct-current cardioversion for torsade de pointes (middle strip), and after successful cardioversion (bottom strip). At baseline (top strip) the ECG demonstrates complete heart block with an uncorrected QT interval of 600 ms. The middle strip clearly demonstrates TdP, which is treated with electrical cardioversion in the centre of the middle strip, followed by return to the baseline dysrhythmia in the bottom strip.

The examination was unremarkable. Resting ECG showed a sinus rhythm at 100 beats per minute (bpm) with complete AV block and a ventricular escape at 33 bpm (Fig. 1, top strip). She was haemodynamically stable and was booked for permanent pacemaker implantation the following day.

At the time of pacemaker implantation, it is usual protocol to give our patients prophylactic antibiotics during and after the procedure. Most patients receive a first-generation cephalosporin in three intravenous doses. However, because this patient had reported an allergy to penicillin, it was decided that an alternative antibiotic be used.

While the patient was being draped for the procedure, a single dose of intravenous erythromycin 500 mg was infused. This was followed by insertion of a transvenous temporary pacing lead via the right femoral vein. The introduction of the transvenous lead into the right ventricle resulted in right ventricular ectopic beats during positioning in the right ventricle, and this resulted in the induction of polymorphic ventricular tachycardia (Fig. 1, middle strip), which required three rounds of DC cardioversion at 200 J over a period of approximately 15 minutes. This restored her to her baseline rhythm (Fig. 1, bottom strip). The patient was transferred back to the coronary care unit where she was given intravenous magnesium and, fortunately, the ventricular tachycardia did not recur.

Cardiac catheterisation performed the following day revealed normal coronary arteries and blood results showed no electrolyte abnormality. Pacemaker implantation was subsequently performed without antibiotic cover and with no further episodes of TdP.

## Discussion

We present a case of TdP due to QT prolongation in a patient with complete AV block who received erythromycin. The patient was not on any other medication known to prolong the QT interval and her serum electrolyte levels were normal. The arrhythmia occurred during insertion of a temporary pacing lead and was treated emergently with electrical cardioversion.

Permanent pacemaker implantation for complete AV block is a common cardiac procedure. Numerous drugs are administered at the time of pacemaker implantation, including antibiotics and anaesthetics. Ginwalla *et al.*,^3^ for example, described a case of haloperidol-induced TdP in a patient with complete AV block. The haloperidol was given to control agitation in an elderly gentleman, which resulted in TdP requiring defibrillation and intravenous magnesium.

Their case and ours highlights the importance of doctors needing to be aware of what medications and electrolyte imbalances to avoid in patients with severe bradyarrhythmias. Our case highlights the care required in the peri-operative patient with complete AV block who may receive a number of potentially hazardous drugs.

It has been known for many years that erythromycin prolongs the QT interval in susceptible individuals and can lead to potentially fatal dysrhythmias.5 The mechanism of QT prolongation due to erythromycin is related to blockade of potassium efflux in the plateau phase of the myocardial action potential.^6^,^7^ Daleau *et al.*^8^ were the first to demonstrate that the effect of erythromycin is to inhibit the rapid component of the delayed rectifier current (I_Kr_) in guinea pig myocytes.

Prolongation of the action potential increases the risk of early after-depolarisations in M cells deep within the ventricular myocardium and creates marked dispersion of repolarisation across the ventricular wall.^7^ This sets the stage for maintaining the characteristic spiraling ventricular arrhythmia, TdP. The development of right ventricular ectopic beats during temporary pacemaker insertion can create the classic ‘short–long’ R–R intervals associated with polymorphic ventricular tachycardia initiation, which is then maintained by the erythromycin-induced dispersion of repolarisation.

The mechanism of QT prolongation in patients with bradycardia-related TdP is poorly understood. Certainly, not all patients with AV block get prolongation of the QT or TdP. Kurita *et al.*^9^ demonstrated that patients with complete AV block with TdP have a bradycardia-sensitive repolarisation abnormality, which persists even after pacemaker implantation. However, there is little data on the actual mechanism responsible for QT prolongation and it may just be that those individuals who develop QT prolongation and TdP have an underlying genetic predisposition, resulting in prolongation of the action potential duration, which is exacerbated by the bradycardia.

## Conclusion

Our case re-emphasises the risks involved in administering erythromycin to patients. In particular, patients with AV block should not receive this drug. The timing of the TdP is interesting because it coincided with the insertion of the temporary pacing lead. It is very likely that the ventricular ectopics induced by the temporary lead in the right ventricular apex precipitated the short–long cycle that led to the TdP. Instead of repeated DC cardioversion, we could have temporarily paced the patient at a more rapid rate to prevent the recurrence of the bradycardiainduced TdP. Based on data from Kurita *et al.*,9 a rate above 60 bpm would have been sufficient to prevent further episodes of TdP.
